# Effect of Application of a Bio-Adhesive on the Removal Torque Value and Rotational Misfit at the Implant–Abutment Junction: An In Vitro Study

**DOI:** 10.3390/ma14226832

**Published:** 2021-11-12

**Authors:** Mahnaz Arshad, Sina Khayat Zadeh, Mohammad Atai, Gholamreza Shirani, Georgios E. Romanos, Seyed Hossein Bassir

**Affiliations:** 1 Dental Research Center, Dentistry Research Institute, Tehran University of Medical Sciences, Tehran 14155-6447, Iran; arshad-m@tums.ac.ir; 2 Department of Prosthodontics, School of Dentistry, International Campus, Tehran University of Medical Sciences, Tehran 14399-55991, Iran; 3 School of Dentistry, Tehran University of Medical Sciences, Tehran 14399-55991, Iran; sinakhayatzadeh@yahoo.com; 4 Iran Polymer and Petrochemical Institute (IPPI), Tehran 14977-13115, Iran; m.atai@ippi.ac.ir; 5 Department of Oral and Maxillofacial Surgery, School of Dentistry, Tehran University of Medical Sciences, Tehran 14399-55991, Iran; shirani@sina.tums.ac.ir; 6 Department of Periodontology, School of Dental Medicine, Stony Brook University, Stony Brook, NY 11794, USA; georgios.romanos@stonybrookmedicine.edu

**Keywords:** adhesives, dental implants, dental implant–abutment design, in vitro, torque

## Abstract

The aim of this study was to assess the effect of application of a recently developed bio-adhesive (Impladhesive) to abutment screw threads on the removal torque value and rotational misfit at the implant–abutment junction. This in vitro study evaluated 20 implant fixtures and 20 straight abutments. Specimens were randomly divided into two groups (*n* = 10) with/without adhesive application. In the adhesive group, the abutment was dipped in Impladhesive before torquing. In the control group, the abutment was torqued conventionally without adhesive application. The removal torque value was recorded after completion of the cyclic loading of 500,000 cycles with 2 Hz frequency and 75 N load. Rotational misfit was recorded using a video measuring machine. After applying the torque, the change in the bisector angle on the abutment hex was recorded for each implant. The biocompatibility of Impladhesive was evaluated using a MTT cell vitality assay. Normal distribution of data was assessed using the Kolmogorov–Smirnov test. Data were analyzed using a *t*-test and Pearson’s correlation coefficient The application of Impladhesive at the implant–abutment interface resulted in significantly greater mean removal torque value compared to the control group (*p* = 0.008). In addition, the mean rotational misfit at the implant–abutment interface was significantly lower in the use of Impladhesive compared to the control group (*p* = 0.001). In addition, the cell vitality was found to be greater than 80% at all evaluated time points. It can be concluded that the application of Impladhesive on the abutment screw significantly decreased rotational misfit and increased the removal torque value. Future studies are needed to evaluate the efficacy of this bio-adhesive an in vivo setting.

## 1. Introduction

Replacement of the missing teeth by implant-supported restorations is a predictable treatment modality [1,2]. The outcomes of implant therapy have been greatly enhanced by technological advances in implant surface materials, surgical techniques, and the stability of interfaces between different components such as the implant fixture, abutment, and prosthetic restoration [3,4]. However, some cases of failure are still reported due to some technical and/or biological complications [3]. After loading of an osseointegrated implant, the occlusal functional loads are transferred to the implant through the implant–abutment junction. Any deformation or wear of prosthetic restoration due to misfit can compromise the long-term stability of this junction and lead to technical complications, such as abutment screw loosening [5].

Abutment screw loosening is a common complication of dental implant restorations that affects their long-term clinical outcomes, and this can lead to the abutment screw fracture or even biological complications such as peri-implantitis [5,6]. Preload and removal torque value are two important variables that influence the screw loosening. Preload is a tensile load that is generated when tightening the abutment screw into the implant fixture and is required to maintain the assembly of the components [7]. Higher preload increases the resistance of the screw to loosening and further stabilizes the junction [8]. Application of external loads to the abutment that exceed the preload leads to micro-movements and instability of the abutment [9]. Several variables can affect the magnitude of this load, such as the torque applied for abutment tightening, type and design of the implant–abutment junction, screw design, screw material properties, microstructure and microscopic irregularities of the implant–abutment interface, the magnitude of applied load, presence of lubricants, and contamination with saliva and oral debris [10]. Higher torque applied for abutment tightening would result in higher preload [11]. The removal torque value is the magnitude of rotational force required for the retrieval of the abutment screw from the implant fixture. The magnitude of removal torque value is usually 80–90% of the torque applied for abutment tightening [12]. The higher the share of removal torque value from the primary torque value, the lower the risk of screw loosening would be. The removal torque value and the preload would decrease following the occurrence of microleakage as a result of misfit between different implant components [13]. Several strategies have been proposed to increase the preload and prevent screw loosening, such as the use of anti-rotation inlay in the screw access hole [14], creation of a bar for mechanical retention of the screw in the screw hole [15], or mechanical alterations of the screw access hole [16]. Our group has previously showed that the application of an industrial adhesive material around the screw of the abutments can significantly increase the removal torque value and reduce screw loosening [17]. However, the adhesive that was used in the previous study was an industrial adhesive with limited biocompatibility. Hence, engineering a biocompatible adhesive can be a viable option to reduce the chance of the screw loosening.

A precise fit of the abutment to the implant is crucial for the success of implant therapy [18]. Misfit between different implant components, especially the abutment and fixture, can negatively affect the long-term stability of dental implants and lead to biological and mechanical complications, such as screw loosening and peri-implantitis [13]. It has been shown that the stability of the abutment screw is significantly affected by the rotational freedom of the abutment [19,20]. The rotational misfit of the abutment in the fixture can significantly increase the risk of screw loosening if it is more than 5° [19]. Hence, improving the rotational misfit can reduce the possibility of the abutment screw loosening.

The aim of this in vitro study was to evaluate the effects of application of a newly developed bio-adhesive (Impladhesive) [21] to abutment screw threads on the removal torque value and rotational misfit at the implant–abutment interface. In addition, the biocompatibility of this bio-adhesive was assessed using a cell vitality assay. This bio-adhesive is based on methacrylate molecules including Bis-GMA, TEGDMA, and methyl methacrylate (MMA), where Bis-GMA and TEGDMA function as crosslinking agents, and MMA is a reactive diluent. This self-cured acrylate-based bio-adhesive was applied on the abutment screw prior to placing them in the fixture and torquing. The null hypothesis was that the application of Impladhesive does not significantly affect the removal torque value and rotational misfit.

## 2. Materials and Methods

The study protocol was reviewed and approved by the ethics committee of Tehran University of Medical Sciences (IR.TUMS.DENTISTRY.REC.1397.180).

### 2.1. Sample Size Determination

Power calculation was done using the one-way ANOVA power analysis option of PASS II software (NCSS, LLC, Kaysville, UT, USA), with the setting of beta = 0.2, alpha = 0.05, a standard deviation of 15 [22], and effect size equal to 0.57. The sample size for each group was determined to be 10.

### 2.2. Adhesive Preparation

A monomer mixture containing 50 wt % Bis-GMA (2,2-bis[4-(2-hydroxy-3-methacryloxypropoxy) phenyl] propane, Evonik, Essen, Germany), 20 wt % TEGDMA (Triethylene glycol dimethacrylate, Evonik, Essen, Germany), and 30 wt % MMA (methyl methacrylate, Sigma-Aldrich, Steinheim, Germany) was prepared. Then, the mixture was divided into two equal parts. In the first part, 0.5 wt % benzoyl peroxide (BP, Merck, Darmstadt, Germany), as initiator, was incorporated and in the second part, 0.5 wt % N,N-dimethyl-p-toluidine (Sigma-Aldrich, Steinheim, Germany) was added as amine activator.

Prior to application, these two parts are mixed, and then, the mixture is applied on the screws. On mixing, the BP/amine redox system is activated and initiates the free radical polymerization of the methacrylate monomers. The polymerized adhesive seals the screw gaps and stabilizes the screw.

### 2.3. Sample Preparation

**Figure 1:**
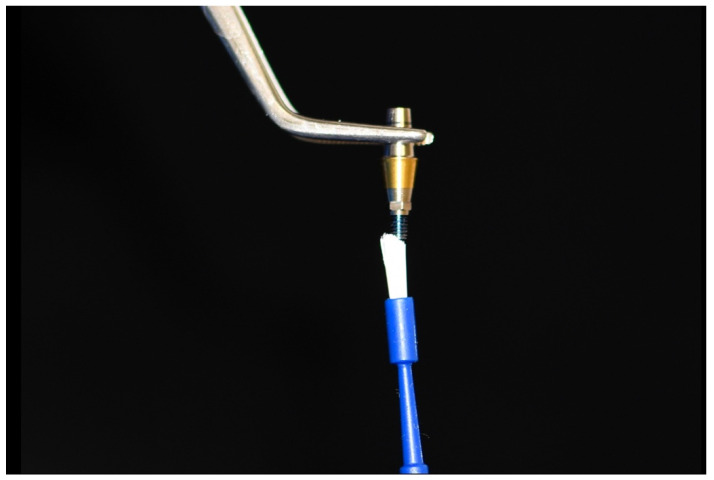
Application of Impladhesive bio-adhesive to the abutment screw threads.

**Figure 2:**
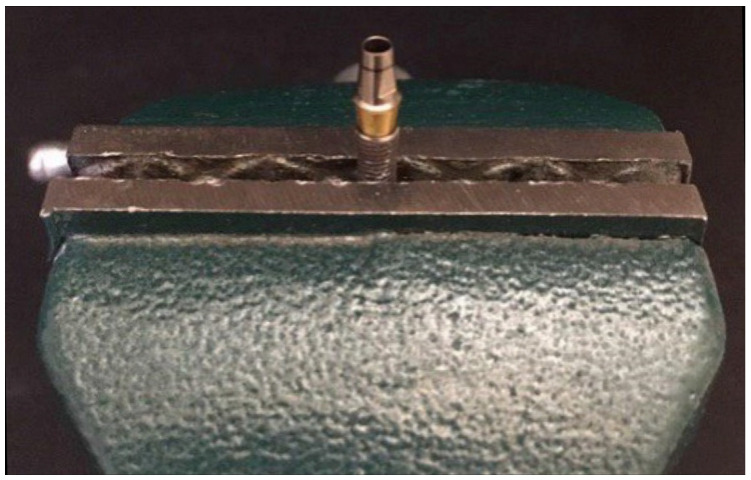
Placement of the abutment–implant assembly into the fixation clamp.

A total of 20 implant fixtures (SuperlineFX 4012 SW, Dentium, Seoul, Korea) with a 4 mm platform diameter and 12 mm height as well as 20 straight abutments (Dual abutment DAB 45 35 HL, Dentium, Seoul, Korea) with 4.5 mm diameter, 5.5 mm height, and 1.5 mm gingival height were used in this in vitro experimental study. The abutments were connected to the implants and coded. The random allocation of specimens to the test group (with adhesive) or control groups (without adhesive) was determined by flipping a coin. A holding device was set up on a table, and its stability was ensured. In the adhesive group, Impladhesive was applied to the abutment screw threads (Figure 1). A trained operator placed the abutment screw in each implant fixture. Another trained researcher blinded to the group allocation applied the torque. Each abutment–implant assembly was first fixed to the clamp (Figure 2), and then, the abutment screw was torqued to 30 N·cm using a screwdriver and a torque meter (Lutron Electronic Enterprise Co., Taipei, Taiwan). To benefit from the preload, 10 min were allowed, and the specimens were torqued again to 30 N·cm. Twenty-four hours were allowed for final setting of the adhesive, and then, the specimens underwent cyclic loading.

### 2.4. Fabrication of Crown for Cyclic Loading

Crowns were fabricated according to a previously published methodology [17]. Crowns with a 45° angle were fabricated from base metal alloy (Wirobond C; Bego, Bremen, Germany). The crowns were formed uniformly and had a horizontal bolt for enhanced retrieval after cyclic loading. When load is applied at a 45° angle, it is split into horizontal and vertical vectors to the specimens. The crowns were placed over the abutments without temporary or permanent cementation because they had adequate stability. This allows for removal of crowns with ease and without any damage after cyclic loading [17].

### 2.5. Cyclic Loading

Cyclic loading was performed according to a previously published methodology [17]. After 24 h, the specimens were mounted in auto-polymerizing acrylic resin for cyclic loading in a chewing simulator (S-D mechatronic GmbH, Feldkirchen-Westerham, Germany). The fixtures were first wrapped in thin aluminum foil to prevent the leakage of acrylic resin into the fixture threads and to facilitate the separation of fixtures from the acrylic resin after the completion of cyclic loading. Next, the internal surface of the molds of the chewing simulator was lubricated with petroleum jelly. Two layers of modeling wax (Cavex, Haarlem, The Netherlands) were applied at the bottom of the molds to enhance the removal of specimens after cyclic loading. The specimens were embedded in wax using a surveyor to ensure load application along the longitudinal axis of the abutment and implant. Auto-polymerizing acrylic resin (GC Corp., Tokyo, Japan) was applied in the mold to 1 mm below the implant–abutment connection. After completion of the setting reaction, the blocks were mounted in the chewing simulator and subjected to 500,000 cycles with 2 Hz frequency and 75 N load [17], corresponding to one year of clinical service in the oral environment. The tip of the load applying rod was rounded and matched the 45° crown angulation.

### 2.6. Measuring the Removal Torque Value

After completion of the cyclic loading, the specimens were de-torqued using a torque meter and the removal torque value was recorded. To do so, the implants were first fixed to the holding device. Next, the digital torque meter (Lutron Electronic, Taipei, Taiwan) was adjusted to record the maximum torque value. The abutment screw was slightly loosened using the respective screwdriver, and maximum torque applied for opening the screw was recorded as the removal torque value.

### 2.7. Measuring the Rotational Misfit

**Figure 3:**
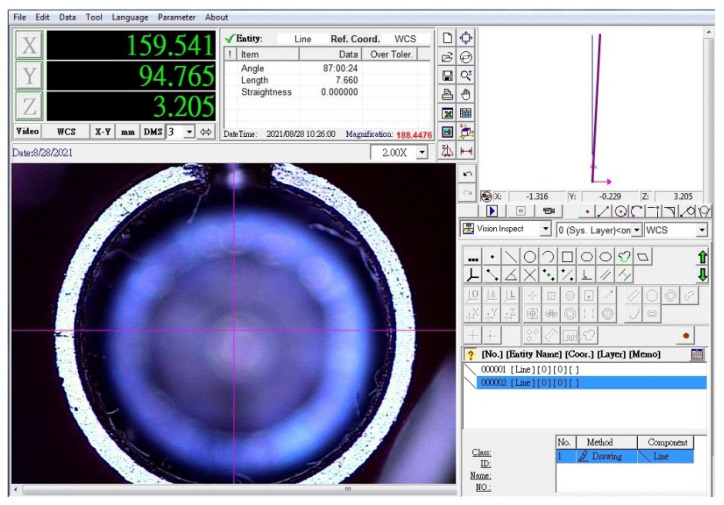
Evaluation of rotational misfit using video measuring machine.

A thin groove was created on the abutment using a disc-shaped bur. The implant was placed in the respective clamp and subjected to the video measuring machine (VMM, ARCS, Taichung, Taiwan). The focus of the VMM was adjusted on the groove site. Next, using a needle holder with locking mechanism, the abutment underwent counter-clockwise rotation until further rotation was impossible. The incision margins were recorded in this position using VMM, and the hypothetical bisector line of the angle between these two lines was drawn. Next, the abutment underwent clockwise rotation, and another image was obtained by the VMM. The difference in the bisector angles between the two steps was calculated and recorded as the rotational misfit (Figure 3). All measurements were made by a single trained individual who was blind to the group allocation.

### 2.8. Assessing the Biocompatibility

The biocompatibility of Impladhesive was assessed using a MTT cell vitality assay [23]. Three test implants where Impladhesive was applied to the abutment screw threads and one control implant without adhesive were included. Test implants each were aged in 2 mL of RPMI culture media for one hour, 24 h, and one week. In addition, the control implant was aged in 2 mL of RPMI culture medium for one week. After each aging interval, the extracts were collected for MTT cell vitality assay.

L929 Fibroblasts were seeded into 96-well plates with 1 mL of RPMI medium per well (14,000–16,000 cells per well) and maintained in the culture medium for 24 h. Then, the medium above cells was removed, and 100 uL of extracts from samples were added to each well. Negative control wells consisted of cells cultures without the extracts. After 24 h, the culture medium was replaced with 100 uL MTT for each well, and cells were incubated for four hours at 37 °C in 5%CO_2_/95% air. The formation of formazan was confirmed under light microscopy. Then, MTT solution was removed, and 100 uL of dimethyl sulfoxide (DMSO) solution was added to each well to solubilize the produced formazan. Cells were incubated for 20 min until all crystals were dissolved. The cell vitality was assessed by measuring the absorbance of cell lysate using a specetophotometer at 540 nm. MTT assays were performed in triplicate.

### 2.9. Statistical Analysis

Data analysis was performed using SPSS version 20 (IBM, Armonk, NY, USA). Normal distribution of data was assessed using the Kolmogorov–Smirnov test. Data were analyzed using descriptive statistics, *t*-test, and Pearson’s correlation coefficient. Statistical significance was set at alpha = 0.05.

## 3. Results

The removal torque values and rotational misfit values for test and control groups are presented in the Table 1.

The removal torque value ranged from 10 to 24 N·cm in the control group with a mean removal torque value of 20.80 N·cm. The mean removal torque value was 26.60 N·cm with a range of 24 to 30 N·cm in the test group. The difference in the mean removal torque between the test and control groups was statistically significant, and the removal torque values were greater in the test group, where the bio-adhesive was used, compared to the control group (*p* = 0.008).

The mean rotational misfit in the control group was 5.29 degrees ranging from 4.98 to 5.59 degrees. The mean rotational misfit ranged from 4.18 to 5.35 degrees in the test group with an average of 4.84 degrees. The statistical analysis showed that the rotational misfit values were significant less in the test group compared to the control group (*p* = 0.001).

Pearson’s correlation coefficient demonstrated that a significant inverse correlation existed between the rotational misfit at the implant–abutment interface and the removal torque value (*p* = 0.024, r = −0.503).

**Figure 4:**
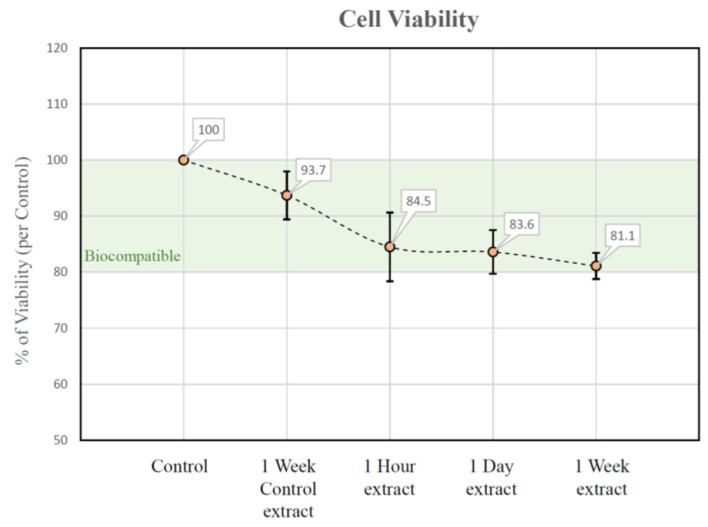
Results of MTT cell vitality assay for negative control, positive control, and test samples after 1 h, 1 day, and 1 week.

The results of cell vitality assay are illustrated in the Figure 4. High cell vitality (>80%) was observed for all evaluated time points, indicating the promising biocompatibility of the Impladhesive bio-adhesive.

## 4. Discussion

The abutment screw loosening is a common postoperative complication of implant-supported restorations [24,25]. The prevalence of torque loss has been reported to range from 19.7% to 39% [26]. Screw loosening leads to instability of the implant–abutment junction and formation of a microgap, which may result in the fracture of implant components [27]. This microgap also enables the bacterial leakage. The main objective of this study was to develop a method to increase the retention and stability of the abutment screw. Thus, Impladhesive was used, which contains methacrylate-based monomers. This adhesive provides a strong adhesion between the screw and implant fixture, seals the screw gaps, and lubricates the screw. In addition, cyclic loading was performed to simulate the loads applied to the fixture–abutment–crown assembly in the clinical setting [17].

The current findings demonstrated that the application of Impladhesive resulted in significantly lower rotational misfit and higher removal torque values compared to the control group, supporting the efficacy of Impladhesive. These findings can be attributed to the wetting property of the adhesive, its adhesiveness, and overcoming the friction force. These results are in agreement with the result of our previous study where it was found that the application of an industrial adhesive (Loctite, Henkel Adhesives Technologies, Rocky Hill, CT, USA) on the abutment screw significantly increased the removal torque value [17]. In line with the current study, Jank et al. (2005) used saline, petroleum jelly, chlorhexidine gel, and Listerine mouthwash as lubricants at the screw–abutment interface and reported higher preload compared with the control group [28]. Coating of this area with a dry lubricant, such as 60–80 nm titanium nanoparticles, petroleum jelly, and human saliva can decrease friction and increase and preserve the preload by adjusting the leakage effect [29]. The use of Impladhesive can also yield lubricating properties during screw tightening due to its slow setting process.

The microgap at the implant–abutment interface can affect the removal torque value. Nigro et al. compared dry and moist environments during the abutment screw tightening and reported that the removal torque value in the moist (saliva) group was significantly higher than in the dry group even after 10 times of tightening and loosening. They concluded that part of the applied torque for screw tightening is used to overcome the friction, and thus, lubricating the screw with the saliva can decrease friction and increase the preload [30]. Tzenakis et al. (2002) evaluated the effect of re-torquing and saliva accumulation on gold screw preload and showed that the re-torquing of a saliva-lubricated gold screw increased the preload [31]. According to Gross et al., the microgap at the implant–abutment interface can serve as a passageway for liquids and macromolecules present in the saliva or gingival crevicular fluid [32]. The liquids leaked through this microgap may contain molecules that are pivotal for the growth and proliferation of bacteria. These bacteria and their by-products can cause clinical peri-implantitis and malodor. Microleakage can also lead to screw loosening and reduction of removal torque value [13]. Moreover, screw loosening can increase microleakage. These findings indicate that application of a bio-adhesive such as Impladhesive to fill the microgap at the implant–abutment interface might be beneficial not only to increase the removal torque value but also to decrease the microleakage and risk of developing peri-implant diseases.

Misfit at the implant–abutment interface in two-piece dental implants leads to bacterial colonization at the interface and can significantly compromise the implant success [33,34]. The microgap between the abutment and implant inevitably exists. Although the size of bacteria ranges from 0.5 to 2 µm, a gap size up to 51 µm between the abutment and implant is acceptable [35]. Thus, adhesive materials can be applied to fill the gap at the interface and enhance the retention of the abutment screw [17]. Moreover, filling the annulus by the adhesive would eliminate the risk of accumulation of bacteria in this space and their subsequent leakage through the implant–abutment microgap as the result of pumping movement of the abutment crown. By doing so, the risk of crestal bone resorption would be minimized, especially early after crown delivery [17].

The strength of the present study was that a novel self-cured acrylate-based bio-adhesive was used on the abutment screw, and it was shown that this bio-adhesive can significantly improve the removal torque and rotational misfit of the implant–abutment interface. Nevertheless, the limitations of the present study should be considered while interpreting the results. The shape and configuration of the implant–abutment interface can affect the rotational misfit and removal torque value [36]. In the present study, both test and control groups had the same implant–abutment interface. However, the efficacy of the Impladhesive bio-adhesive should be also investigated in different implant–abutment designs. In addition, another limitation of the present study is the in vitro setting of the study, which may not necessarily replicate the intraoral environment. Considering the promising outcomes on this in vitro study, the next step would be assessing the efficacy and biocompatibility of the Impladhesive bio-adhesive in an in vivo setting. Furthermore, the effect of application of the bio-adhesive on the other outcome variables such as the chance of screw fracture should be assessed.

## 5. Conclusions

Within the limitation of this study, it can be concluded that the application of the Impladhesive bio-adhesive at the implant–abutment interface significantly decreased the rotational misfit and increased the removal torque value. This may result in preventing screw loosening or decreasing the frequency of screw loosening. Considering the promising biocompatibility of this bio-adhesive, future studies are warranted to evaluate the efficacy of this bio-adhesive an in vivo setting.

## Figures and Tables

**Table 1:** Measurements of removal torque value (N·cm) and rotational misfit (degree) at the implant–abutment interface in the test and control groups (*n* = 10).

Variable	Groups	Mean	SD	*p*-Value
Removal torque value	Control (no adhesive)	20.80	4.158	0.008 *
	Test (with adhesive)	26.60	1.838	
Rotational misfit	Control (no adhesive)	5.293	0.148	0.001 *
	Test (with adhesive)	4.842	0.414	
	Test (with adhesive)	4.842	0.414	

* Statistically significant difference between the two groups. SD = Standard Deviation.

## Data Availability

Not applicable.
